# Viability of Probiotic Microorganisms and the Effect of Their Addition to Fruit and Vegetable Juices

**DOI:** 10.3390/microorganisms11051335

**Published:** 2023-05-19

**Authors:** Maria Spinasse Maia, Manueli Monciozo Domingos, Jackline Freitas Brilhante de São José

**Affiliations:** 1 Integrated Health Education Department, Federal University of Espírito Santo, Maruípe Campus, Marechal Campos Avenue, Vitória 29040-090, ES, Brazil; 2 Postgraduate Program in Nutrition and Health, Federal University of Espírito Santo, Maruípe Campus, Marechal Campos Avenue, Vitória 29040-090, ES, Brazil

**Keywords:** probiotics, food science, non-dairy products, functional foods, human health

## Abstract

Consumers’ recent interest in healthier diets has increased the demand for food products with functional properties, such as probiotics. However, most probiotic food types available on the market are of dairy origin, which limits their consumption by individuals with food intolerances and by those who adhere to strict vegan and vegetarian diets. The aim of the current review is to assess both the limitations and impacts of the addition of probiotic microorganisms to fruit, vegetable, and/or mixed juices. Thus, an integrative literature review was herein carried out. A bibliographic survey was carried out in the following databases: Lilacs, Medline, Web of Science, Scopus, and Scielo. In addition, searches for studies published in English from 2010 to 2021 were carried out, based on the following meshes: “fruit”, ‘‘vegetable”, ‘‘juice”, and “probiotics”, which were used both in combination with each other and with Boolean operators such as “AND” and “OR”. Although 254 articles were initially found in the literature search, only 21 of them were selected to compose the final sample. The included studies mainly addressed microorganism viability and physicochemical analyses. Overall, fruit and/or vegetable juices can be suitable matrices used to help the development of probiotic food types. However, the microorganisms added to these products must be capable of adapting to and surviving in them to enable a product’s success. Therefore, factors such as pH, fiber content, amino acids, and phenolic compounds play an essential role in the survival of probiotic microorganisms. Given the wide variety of analyses, a comparison between parameters was the major limitation of the present study. Future studies should focus on filling the gaps persisting in the development of probiotic fruit and/or vegetable juices as well as mixed juices.

## 1. Introduction

In recent years, there has been an increase in the consumption of fruits and vegetables, mainly due to their health benefits. China is the largest producer of fruits, followed by India and Brazil [1]. The presence of bioactive compounds such as carotenoids, polyphenols, and vitamins, as well as dietary fiber and minerals, makes these foods important for maintaining a healthy lifestyle [2,3]. Including fruits and vegetables in the diet through juices is a practical way to eat healthy foods [3,4]. New trends in the consumption of natural foods and a healthy lifestyle have boosted sales of fruit and vegetable juices.

The possibility of diversifying raw materials in the preparation of juices has added to consumers’ desire for products that are beneficial to health, directing interest in adding compounds with functional properties. Foods claimed to have functional properties are defined as foods that, in addition to their nutritional values, confer benefits on body functions and have become increasingly popular [5].

Consumers’ recent interest in healthier diets has increased the demand for food products with functional properties, i.e., those presenting at least one nutrient or non-nutrient with positive effects, either metabolic or physiological, on human body development and maintenance, among other functions. Fruit juices are potential matrices for the insertion of probiotics since they have several nutritional characteristics favorable for growth while also meeting consumer needs for more natural and healthier foods [6,7].

Probiotic food types fall into this group; moreover, they are associated with several advantages, such as improving intestinal health and treating diseases such as obesity and type 2 diabetes [8,9,10]. According to the scientific literature, when inserted into food, the microbial cultures used must have counts of 10^8^ to 10^9^ cells per gram of product [11,12,13].

The cultures of microorganisms most used for producing probiotic foods may be lactic acid bacteria (LAB) of the genera *Lactobacillus*, *Lacticaseibacillus*, *Lactiplantibacillus*, *Limosilactobacillus* and *Bifidobacterium*, such as the species *Lactobacillus acidophilus*, *Lactobacillus delbrueckii* subsp. *bulgaricus*, *Lactobacillus delbrueckii* subsp. *lactis*, *Lacticaseibacillus casei*, *Lacticaseibacillus paracasei*, *Lacticaseibacillus rhamnosus*, *Lactiplantibacillus plantarum* subsp. *plantarum*, *Limosilactobacillus reuteri*, *Limosilactobacillus fermentum*, *Bifidobacterium longum*, *B. bifidum*, *B. infantiles*, *B. breve*, *B. animalis*, *B. lactis*, and species from other genera, such as *Streptococcus thermophilus*, *Streptococcus* spp., *Enterococcus faecium* and *Saccharomyces* cerevisiae [14]. It is important to note that there was a reclassification of the genus Lactobacillus, with the definition of 23 new genera based on phenotypic, genotypic, and ecological characteristics, to define in a more delimited way the specificities and characteristics of each group contained in this old genus [15].

Probiotic microorganisms are those that when administered in adequate amounts can have beneficial health effects on hosts [16]. Thus, these microorganisms can be added to food items to help develop products with probiotic properties. However, probiotic microorganisms must survive storage, either in capsules or in food, as well as be consumed on a regular basis to provide the desired benefits. Furthermore, it is worth emphasizing that probiotics must also survive their passage through the gastrointestinal tract (GT) [17].

On the other hand, most probiotic food types traded today are of dairy origin. However, intolerance associated with the intake of milk and dairy products, as well as the increased number of individuals adhering to a vegan lifestyle and strict vegetarian diets in recent years, may limit their consumption. Thus, fruit and vegetable-based products can be an alternative to help overcome this issue [18].

It is essential to point out that the food matrix and the food administration form can also influence the survival and multiplication of probiotics, as well as possibly helping to maintain the viability of microorganisms during the product’s shelf life [17,18,19]. Thus, the digestion of liquid food is faster and reduces their contact time with bile acids and low stomach pH, a fact that enables microorganism cultures to develop resistance to these adverse conditions [20].

The intake of mixed juices, as well as fruit and vegetable juices, has great potential to increase both in Brazil and abroad [21,22]. These drinks hold significant amounts of vitamins, minerals, fiber, antioxidants, and bioactive compounds. Thus, in addition to the increasing consumption of these nutrients by the population, juices also meet the emerging demand for healthier and more natural products [20].

From this perspective, some authors have investigated the use of fruit and/or vegetable juices as potential matrices to help develop probiotic food types [7,8,9,10,11,12,13,14,15,16,17,18,19,20,21,22,23]. However, these products have some stressors capable of hindering the adaptation and survival abilities of microorganisms, namely low pH, oxygen level, antimicrobial components, and storage temperature [24].

**Figure 1:**
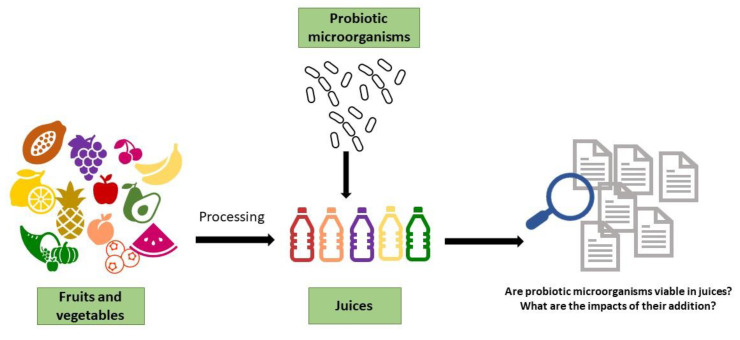
The addition of probiotic microorganisms in fruit, vegetable and/or mixed juices.

Given the increased demand for and benefits resulting from the intake of probiotic food types, as well as the challenges and advantages assumingly associated with the development of beverages derived from non-dairy matrices (Figure 1), the aim of the current study was to assess the viability of probiotic microorganisms and the impacts of their addition to fruit, vegetables, and/or mixed juices based on an integrative literature review.

## 2. Materials and Methods

An integrative review study was conducted based on the method suggested by Souza et al. [25]. This was split into six stages, namely: guiding question elaboration; inclusion and exclusion criteria establishment and search in the literature; and definition of information to be extracted from the selected studies, including study assessment, interpretation of results, and review presentation.

Firstly, the following guiding question was defined: what are the limitations and impacts of adding probiotic microorganisms to fruit and vegetable juices? Then, data collection was carried out, and databases, search strategies, and inclusion and exclusion criteria were defined.

A bibliographic survey was carried out in July 2021 through an electronic search performed in the following databases available in the Virtual Health Library: Lilacs, Medline, Web of Science, Scopus, and Scielo. Health Sciences Descriptors (DeCS) and Medical Subject Headings (Mesh) were used as strategies to search for articles. Boolean operators “and” and “or” were used with keywords “fruit”, “vegetable”, “juice”, “probiotics”, as well as with combinations of them.

The inclusion criteria comprised original articles published in English between 2010 and 2021 that addressed the limitations and impacts of adding microorganisms to fruit and vegetable juices at the time to develop new products. Exclusion criteria comprised review articles, book chapters, editorials, letters to the editor, and studies that did not address the topic associated with the purpose of the current review. Publications available in more than one database were only considered once.

**Figure 2:**
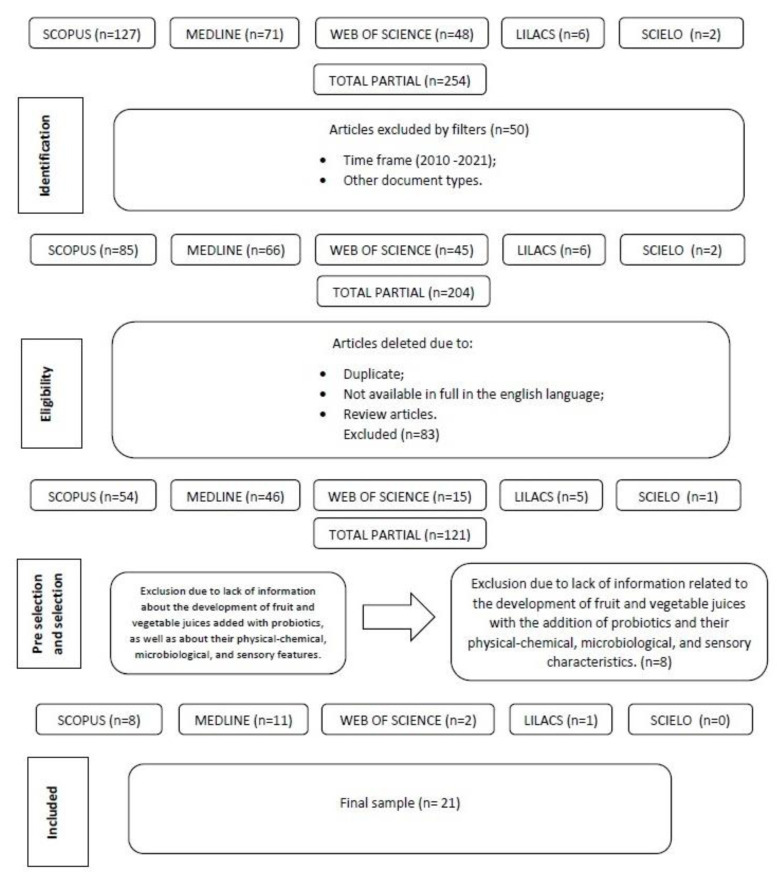
Flowchart showing article selection steps.

The initial search in the literature resulted in 254 articles associated with the herein selected keywords and descriptors. Then, articles published in duplicate were excluded in compliance with the adopted criteria. After the exclusion procedure was completed, 121 studies were considered eligible for the review. They were subjected to a pre-selection stage, according to which their title and abstract were assessed. Whether the selected studies were linked to the current research’s guiding question was also assessed at this stage. After the pre-selection process was completed, 29 articles were identified and assessed to check whether they provided information about the development of fruit and vegetable juices added with probiotics, as well as about their physicochemical, microbiological, and sensory features. After conducting a careful and objective assessment, 21 articles were selected and thoroughly evaluated. They were organized based on the categories of collected information (title, author, year, journal, sample, material and methods, and main results). A flowchart of the steps taken from the bibliographic survey to article selection is shown in Figure 2.

Data were collected from the selected studies, grouped in charts, tables, and thematic approaches, and evaluated and interpreted based on the literature. Finally, the results and discussion were herein presented and organized to help form a better understanding of the topic of this review.

## 3. Results

Of the 254 initially identified articles, 21 were included in the current review. Six (6; 2.4%) of them were indexed in the Lilacs database; 71 (27.9%) in Medline; 127 (50%) in Scopus; 48 (18.9%) in Web of Science; and two (0.8%) in Scielo.

### 3.1. Main Juice-Featuring Analyses

The herein selected articles mainly addressed the viability and physical-chemical analyses of microorganisms (Table 1). The most performed analyses comprised viability tests, which were carried out in 21 studies included in the current review. They were followed by pH (n = 17), total titratable acidity (n = 10), total soluble solids (n = 8), organic acids (n = 6), total sugars (n = 6), instrumental color (n = 6), sensory (n = 5), antioxidant capacity (n = 5), phenolic compounds (n = 5), microbiological (n = 4), and reduced sugars/carbohydrates (n = 3) analyses. However, other juice features were analyzed in less than three of the selected publications, namely: quantification of macronutrients (lipids, proteins, and carbohydrates) [26], the formation of volatile compounds [10,27], and amino acids [27], anthocyanin concentrations [26,28], viscosity [29], turbidity [30], and fiber [31] and inulin contents [32].

From this perspective, there is no standard in the analyses carried out in studies that investigate the development of fruit and vegetable juices added with probiotic microorganisms. This fact limits likely comparisons between results and the formation of a consensus in the literature about the physicochemical aspects associated with developing fruit and vegetable juices with these microorganisms. Thus, any innovative product should be subjected to an in-depth assessment to help fill the gaps associated with the impacts of adding probiotics to plant-origin products. On the other hand, it is essential to emphasize that specific analyses, such as those focused on measuring the amount of inulin added to juices and anthocyanin concentration, were mentioned in some studies. Still, their inclusion depends on the research type and the assessed food matrix.

Thus, studies need to be planned to consider each raw material used, the conditions of inoculation of probiotic microorganisms, and physical-chemical, microbiological, sensory, and nutritional parameters. With all this information, it is possible to analyze more deeply the impacts of the addition of microorganisms to vegetable matrices such as fruit and vegetable juices.

### 3.2. Juices Used in Studies

With respect to juice featuring, fruits were the main matrices used to prepare the juices assessed in the analyzed studies. The main fruit used was the orange, being used as a food matrix in six studies, followed by apple, pomegranate, and grape, used in five, four and two studies, respectively. Only two studies used vegetables to prepare juices: pumpkin [39] and Chinese jujube [10]. Orange is an important fruit recognized for its sensory and nutritional characteristics and health benefits, as well as being an important source of vitamins, mainly vitamin C, fiber, and minerals. It has high antioxidant capacity due to its high content of bioactive compounds such as ascorbic acid, flavonoids, and carotenoids [1,22,27,43].

Mixed juices presented different formulations comprising different fruit and vegetable types, which were combined with each other at different proportions during preparation. This result may be associated with the variety of and accessibility to fruits and vegetables since the surveys were conducted in different countries at different times. Mixed juices have been explored as an alternative to replace ultra-processed beverages and are a convenient way to consume products with high nutritional value [31].

### 3.3. Types and Number of Probiotic Microorganisms Used in Juices

All selected articles used bacteria as probiotic microorganisms for research purposes; *Lactobacillus* was the main tested genus (Table 1). This bacterial genus encompasses Gram-positive microorganisms naturally found in human GT. *Lactobacillus* strains are mostly used because they are generally recognized as safe (GRAS), as well as because of their benefits and suitability, not only in terms of origin, safety, and resistance but also in terms of growth properties in vitro and during processing, and because of their functional features [40]. According to Frakolaki et al. [46], the strains must endure the acid gastric juice, the bile, and the pancreatic enzymes in order to reach the small intestine. Then, they must be able to adhere to the intestinal surfaces. Furthermore, the strains need appropriate technological characteristics, as resistance to aerobic conditions and could be product in industrial scale.

In addition to these factors, probiotic strains need to be storage stable, present satisfactory counts and not interfere with the sensory attributes of the products. With so many necessary factors to be met, it is clear that developing probiotic food is a challenge.

Some of the selected articles included other bacterial genera but the authors considered them as probiotics. There is often some confusion in defining a strain as a probiotic. It is important to highlight that there is a difference between commensal and probiotic microorganisms. Commensal microorganisms in the gut are usually sources of probiotic strains but they cannot be called “probiotics” because the strains still need to be isolated and their possible health effects characterized and evaluated. Another problem is considering live cultures present in fermented foods as probiotic [16].

With respect to the microorganisms’ inoculation form applied to juices, five studies assessed the addition of bacteria in the microencapsulated form. These studies aimed at comparing the impacts of adding microorganisms to juices in their microencapsulated and free forms. According to Rengadu et al. [44], microencapsulation is an efficient method to assist in the protection of probiotics. This technique enables it to survive in food and during passage through the GT. There are different materials that can be used in the microencapsulation process such as polysacaharides, which can be used in combination with gelatine, alginate carrageenan, and starch. Another advantage to these methods is the fact that microcapsules provide an appropriate anaerobic condition to the probiotic bacteria and work as a physical obstacle to the acidic conditions associated with fruit juice [44]. In addition, it is important that the use of this method does not interfere with the survival of microorganisms and their action.

Although data from the literature indicate that the addition of microencapsulated probiotics to foods is a good proposal, it is worth noting that depending on the material used, dissolution may occur, altering the quality parameters of the juice, including color, flavor, viscosity, and aroma. Then, if the juice matrix is an adequate environment for probiotics, this enables its possible use in free form.

Concerning to the addition of isolated or combined microorganisms, probiotics in juices were individually assessed in some studies, except for the study by Rengadu et al. [44], who evaluated the impact of microorganisms separately, and analyzed the accumulation of *Lacticaseibacillus casei* and *Bifidobacterium animalis* combined in the same sample.

The number of probiotics inoculated in the tested juices ranged from approximately 10^6^ to 10^10^ CFU/mL. According to the criteria established by FAO/WHO [19], these products must hold at least 10^6^ to 10^7^ CFU/mL of microorganisms during their shelf life to be considered probiotic. However, according to the National Health Surveillance Agency in Brazil, the minimum number of probiotics in the daily recommendation of these products must range from 10^8^ to 10^9^ CFU [8]. It is important to note that, depending on the country, the number of probiotic microorganisms may vary according to what the authors consider an appropriate recommendation. This could explain the diversity in the values found regarding the amount of initial inoculum added to the juices.

It is essential highlighting that juices and vegetables are unconventional matrices for probiotic microorganisms, besides having stressors, with emphasis on as low pH. Therefore, microorganism inoculation in amounts slightly above the limits established by the legislation would be an interesting strategy since cultures must adapt to and survive in juices to enable these products to exert their benefits.

### 3.4. Probiotic Microorganisms’ Viability in Juices

The viability of probiotics is directly linked to features of the food matrix and added microorganisms, as well as interactions between them. Although there is no consensus in the literature about the exact number of viable cells necessary to trigger probiotic effects, most studies adopt values ranging from 10⁶ to 10⁷ CFU/mL [26,31,38,45].

Many factors can influence the viability of probiotic microorganisms in food products during production, processing, and storage. The characteristics related to the probiotic microorganisms involved, such as strains and the amount of inoculum used, directly influence their viability. In addition, the characteristics of the food are also important, such as pH, titratable acidity, molecular oxygen, water activity, and the presence of salt and sugar. In addition, the addition of chemical substances such as bacteriocins and artificial flavors and colorings should be considered, as should the conditions applied in processing such as heat treatment, incubation temperature, cooling, packaging type, material and storage methods, and production scale [47].

According to Rengadu et al. [44], low pH, nutrient depletion and lactic acid accumulation during storage time can hinder probiotic bacteria survival and affect their effective dose necessary for consumption purposes. Still, it is essential to emphasize that the metabolic specificities of each microorganism, as well as their ability to adapt to breeders’ stressful conditions, are also crucial factors enabling the survival of probiotics [31]. The pH value directly influences the functioning of enzymes, the stability of molecules, and, consequently, cellular metabolism. The different microorganisms have a maximum, optimum, and minimum pH value to enable their growth. Generally, the presence of organic acids (such as lactic, acetic, formic, and benzoic acids) affects the survival of microorganisms, as they are often found in a non-dissociated form and are thus able to more easily penetrate the cell. Organic acids enter the microbial cell and dissociate, releasing H+ ions and causing significant alterations in cellular functioning and the inhibition of microbial growth [48].

De Oliveira et al. [31] assessed three *Lactobacillus* strains (*Lactiplantibacillus plantarum* LP 299V, *Lacticaseibacillus rhamnosus* GG, and *Lactobacillus acidophilus* LA—14) in mixed mango and carrot juice at different concentrations. According to the aforementioned authors, *L. acidophilus* appears to be more demanding when it comes to pH conditions, oxygen level and nutrient viability, in addition to presenting viability lower than that of other microorganisms. On the other hand, the tested *L. plantarum* strain has shown greater viability because this microorganism is acknowledged for its excellent adaptation to adverse conditions, its high capacity to ferment different sugars, and its efficient transport system. Despite this, all strains tested in the current study have shown viability higher than 7 log_10_ CFU/mL after fermentation and at the end of 35-day storage at 8 °C.

On the other hand, the study by Mokhtari et al. [30] assessed bacteria belonging to different genera (*Lactobacillus acidophilus* and *Bifidobacterium bifidum*). The results show that *L. acidophilus* is more resistant than *B. bifidum* under acidic conditions. Therefore, it presented better compatibility with the investigated conditions (grape juice with pH 3.8 during 8-week storage). However, this difference was not significant, and both microorganisms recorded viability higher than 7.0 log_10_ CFU/mL after 60 days at 4 °C.

Thus, in line with other studies, bacteria belonging to the genus *Lactobacillus* are often resistant and capable of surviving in juices with a pH ranging from 3.7 to 4.3. On the other hand, bifidobacteria are less acid-tolerant and pH values close to 4.6 can be detrimental to their survival. However, as shown in the current review, the aforementioned probiotics, even at lower concentrations, have shown good viability in juices with low pH. In these cases, this parameter alone cannot explain the herein observed trends.

From another perspective, the chemical composition of nutrients, balance, and viability, as well as the presence of inhibitory compounds and intrinsic factors in food, can be decisive features used to select the appropriate matrix to help develop probiotic products. This review has found that six studies assessed the same microorganism in more than one juice. In this way, it is possible to analyze in more detail the impacts of the composition and characteristics of the juices on the survival of probiotic microorganisms.

The study by Bhat et al. [34] assessed the growth of the microorganism species *Weissella* Kimchi R-3 in orange, pomegranate, and carrot juices under different fermentation (72 h/37 °C) and storage (12 days/25 °C or 5 weeks/4 °C) conditions. The results indicated that the growth of microorganisms during fermentation time, as well as their viability under both storage conditions, were higher in carrot juice. The assessment showed that a considerable number of bacteria remained until the end of the storage period. However, the numbers of bacteria in orange and pomegranate juice were undetectable in the second week of refrigerated storage as well as during 3-to-6–day storage at room temperature. Thus, the primary justification for this finding depends on the low pH of these matrices and the temperature conditions. The metabolism of microorganisms is more active at 25 °C, which increases the production of organic acids and the depletion of nutrients. On the other hand, 4 °C is the temperature outside the optimal microorganism growth range; therefore, it is an adverse condition for bacterial growth. The reduced pH value improves the quantity of undissociated organic acids in fermented products and contributes to the bactericidal effect of these substances [47,49].

However, as previously mentioned, in addition to pH, other features of juices can also hinder the survival of probiotic microorganisms in them. For example, Srisukchayakul et al. [37] tested the addition of bacteria, previously adapted to acidic media, to pomegranate, cranberry and lime juices. The results proved that although all three matrices presented a similar pH, microorganisms were less resistant to cranberry and pomegranate juices due to the high concentration of phenolic compounds with antimicrobial action. According to Dinkçi et al. [49], plant-based materials such as fruits and vegetables impact probiotic viability depending on which phenolic compounds are present, the amount, and the proportion of vegetable added to the product. Thus, it is possible that the choice of which raw material to use can directly influence the viability of the chosen probiotic culture.

On the other hand, according to Olivares et al. [38], maintaining the viability of probiotic cultures in fruit juices is a challenging task since these products have high concentrations of dissolved oxygen. It is worth emphasizing that the most used probiotic microorganisms are of the anaerobic and microaerophilic types; therefore, the presence of oxygen in the product at more significant rates can lead to toxicity and viability loss. Thus, the aforementioned authors have pointed out that vitamin C, as an oxygen scavenger, may have a protective effect during the storage period as well as promote a more favorable anaerobic environment (not observed during fermentation) [38]. However, it is necessary to consider that vitamin C is a very unstable compound and long periods of storage can cause its reduction in food. As a consequence, damage to the survival of anaerobic cells may occur.

With respect to matrix composition, De Oliveira et al. [31] assessed different formulations associating mango juice with carrot, in addition to different *Lactobacillus* species. The study indicated higher microorganism viability in juice presenting higher carrot pulp concentration due to its high fiber content. The authors explained that dietary fibers can significantly influence the survival of probiotic microorganisms after processing and during storage time. Soluble fibers can be used as a substrate for the growth of probiotic microorganisms, whereas insoluble fibers can protect these bacteria by acting as a physical barrier. Furthermore, the growth of carrot pulp was also high, a factor that can be considered a bias and that may have provided greater stability for microorganisms in this formulation.

However, Valero-cases and Frutos [32] investigated inulin addition to mixed carrot and orange juice by adding *Lactobacillus plantarum* CECT 220 to it. They observed higher microorganism viability in juices added with inulin after 15-day storage. However, viability began to decrease in juices without fiber addition after this time. This happened due to the lower concentration of monosaccharides in these samples. Therefore, inulin was a carbon source available for the tested strain during storage time, and it may also have protected the used microorganism during refrigerated storage by preventing cell damage, mainly by physically immobilizing inulin-structured cells since this polymer forms aggregates in aqueous media. One of the great doubts regarding probiotic products of plant origin is related to the viability of probiotic cultures in non-dairy matrices. Prebiotics are characterized as non-digestible compounds by the body that remain intact in the colon, serving as a substrate for beneficial microorganisms present in the human microbiota. In this way, they may be able to increase the viability of cultures of microorganisms inserted in food matrices [6,47,49]. As presented in the previous research, prebiotics such as inulin could help in the survival of probiotic microorganisms.

The herein presented results suggest that the viability of the microorganisms resulted from the synergistic and antagonistic actions of different parameters. pH can be detrimental to the viability of microorganisms, for example, but protein and dietary fiber can protect cells from this type of stress. Finally, although acidity is a disadvantage for the survival of probiotics in juices, incorporating probiotic bacteria in fruit juices can help increase their resistance to subsequent stressful acidic conditions, such as those observed in the human gastrointestinal tract [23].

According to Tripathi and Giti [47], food components play significant roles in food, either providing protection, remaining neutral, or causing harm to probiotic viability. In addition, food additives (sugars, sweeteners, salts, aroma compounds, flavoring and coloring agents, and bacteriocins) could significantly influence the development and viability of probiotic bacteria.

### 3.5. Impact of Probiotic Microorganisms’ Addition on Juices’ Quality Features

#### 3.5.1. pH and Total Titratable Acidity

pH is an indicator of juice quality and possible microbiological activity. Thus, evaluating this parameter becomes important to understand the possible impacts of adding probiotic microorganisms to juices.

Most studies consider pH one of the most critical factors affecting the survival of probiotic bacteria since they must resist the acidity of the juices in order to grow. Thus, both the drop in pH and increase in acidity help to prevent the development of unwanted microorganisms during storage time as well as increase the shelf life of juices. However, if the amount of produced acid is too high, it can affect the product’s sensory features and, consequently, its acceptability [29,40,41,44].

Therefore, the results of the assessed studies have shown that all articles performing pH and acidity analyses during fermentation reported reductions and increases in these parameters, respectively. According to these studies, these findings were associated with the metabolism of microorganisms since they use carbohydrates found in food matrices as an energy source as well as synthesized organic acids.

Moreover, 9 of the 11 articles that carried out pH analyses during storage time also observed a pH decrease in at least one of the tested juices. The explanation for this finding was the same as previously cited. Zhu et al. [42] in turn have also highlighted that an increase in acidity and decrease in pH may have taken place due to juice sugar hydrolysis induced by enzymes released from dead probiotic cells.

However, divergent results were found in the study by Bonaccorso et al. [43], according to whom pH remained constant for 35-day storage at 5 °C. According to the aforementioned authors, it may have happened due to the reduced metabolism of these bacteria at low temperatures. In addition, Majeed et al. [33] evaluated the pH of apple juice added with *Bacillus coagulans* MTCC 5856 for 6 months under refrigeration (4–6 °C), and they did not observe changes in this parameter; the authors also used the justification associated with refrigeration temperature to explain this finding.

Furthermore, Garcia et al. [29] assessed five *Lactobacillus* strains in apple, grape or orange juices kept under refrigeration at 4 °C for 21 days (each strain in one sample). The authors did not observe changes in pH and acidity until the 14th day of storage. In addition, pH in apple juice increased on the 21st day of storage, regardless of the strain or microorganisms’ proliferation, as well as in grape juice, depending on the added culture (Table 1).

Changes in pH and acidity values can be caused by different factors, as suggested by the aforementioned authors. Thus, it is necessary to know the initial pH of the matrix used and, after inoculation, monitor possible changes during storage. Additionally, changes in pH and acidity values may also occur depending on the strain used and its concentration added to the product.

#### 3.5.2. Total Soluble Solids, Organic Acids, Reducing Sugars/Carbohydrates

Total soluble solids are an essential quality parameter for the development of new products. This parameter mostly represents the sugar content as well as a small portion of soluble proteins and amino acids, among other organic materials. Thus, the value of soluble solids is likely to affect the product’s taste since it can indicate its sweetness level [50].

Only 3 of the 8 articles analyzing total soluble solids have analyzed this parameter during fermentation time; two of them found a decrease in this quality parameter. The other five studies analyzed total solids during storage time; half of them did not find significant differences in these values. According to De Oliveira et al. [31], the maintenance of total soluble solid values may be associated with the action of microorganisms in the hydrolysis of insoluble sugars, a fact that promoted balance in this parameter during storage time.

However, Garcia et al. [29] observed different results for total soluble solids. Total solids content increased in apple juice added with *L. brevis* 59, *L. fermentum* 111, and *L. pentosus* 129 and decreased in all orange juice samples during the 21-day storage. According to the aforementioned authors, the decrease observed in this parameter was associated with microorganisms’ consumption of matrix sugars, whereas the increase observed in the hydrolysis of sugar was associated with enzymes released from dead *Lactobacillus* cells.

As previously mentioned, probiotics can metabolize sugars in juice during fermentation and form organic acids. The produced acids can also work as critical secondary carbon sources for microorganisms. Therefore, six articles have evaluated organic acid levels in juices.

The results reported by Wu et al. [28] suggested a decreasing trend in pyruvic, shikimic, citric, and malic acid contents as well as an increasing trend in lactic acid content and blueberry and blackberry juices during fermentation time. These authors advocated that potential probiotics (*S. thermophilus* and *L. plantarum*) can biotransform malic or pyruvic acid into lactic acid or into other products. Furthermore, probiotic microorganisms can also degrade citric acid to produce lactic and acetic acids and diacetyl. Li et al. [10] and de Garcia et al. [29] also observed the same trend towards increased lactic acid, decreased citric acid, and malic acid biotransformation.

Wu et al. [28] and Garcia et al. [29] also associated viable microorganism counts with organic acids’ metabolism. Thus, microorganisms with higher viable counts presented the best enzymatic activity and, consequently, the highest consumption of sugar and the most significant lactic acid formation.

All studies focused on analyzing reducing sugars, and total sugars reported a decrease in these parameters after fermentation. This finding is explained by the consumption of these nutrients by probiotic microorganisms, which used them as an energy source to grow in juices. The total soluble solids content reflects the flavor and other sensorial substances that are characteristic of the juice. The presence of sugars can influence consumer preference and may lead to acceptance or rejection and is considered a determining factor for turbidity. Thus, changes in these parameters can directly impact the acceptability of juices added with probiotic microorganisms.

#### 3.5.3. Phenolic Compounds and Antioxidant Capacity

Beneficial effects attributed to phenolic compounds are associated with their antioxidant activity. Fruits, vegetables, and derivatives of them are the primary source of antioxidants in the human diet. However, phenolic compounds can be altered during processing, storage, and fermentation time [51].

Although several studies have already investigated the effect of fermentation on the quality and functionality of fruit and vegetable juices, information in the literature about changes in their phenolic profiles and antioxidant capacity remains scarce [10,23]. Therefore, five studies performed this analysis to help better understand the effect of these compounds on the survival of strains as well as the effect of probiotics on the phenolic profile of the juices [10,26,28,36,51].

It is important to emphasize that phenolic compounds play an essential role in the viability of probiotic microorganisms [23]. Valero-cases et al. [35], for example, stated that probiotic bacteria’s growth in pomegranate juice was associated with the metabolism of most phenolic compounds found in this fruit to a greater or lesser extent, depending on the used strain.

On the other hand, Almeida Bianchini Campos et al. [26] have shown that total phenolic compounds’ content was higher in the control juice than in the fermented one. According to these authors, this finding may be explained by the decrease in pH since the co-pigmentation of these compounds is favored by medium acidification. However, anthocyanin content did not change after fermentation since these flavonoids remain stable in acidic media.

Similarly, Mustafa et al. [36] assessed the behavior of phenolic compounds in pomegranate juice at different fermentation temperatures (30 °C, 35 °C, and 37 °C). The results indicated increased phenolic acid and flavonoid contents after 24-h of fermentation. These changes were associated with the used strains and the temperature conditions the samples were exposed to. The variation in the content of free phenolic compounds in fermentation may be related to the action of microbial enzymes that attach glycosidic bonds to these compounds. Considering that each microorganism has an enzymatic profile, different impacts can be observed, and this generates unique flavors and products after the fermentation process. Thus, fermentation with different species of microorganisms causes different enzymatic reactions, followed by different releases of phenolics from the cell wall of food matrices [36].

Wu et al. [28] observed significant changes in phenolic compounds in black and blueberry juices after 48-h of fermentation. Both juices presented similar variations in some phenolic acids. Chlorogenic acid and procyanidin contents decreased, whereas gallic, caffeic, syringic and ferulic acid contents increased. According to the aforementioned authors, anthocyanins in juices may have been degraded into syringic, ferulic and gallic acids. Furthermore, probiotics may have metabolized chlorogenic acid into caffeic acid during fermentation time.

Based on the study by Wu et al. [28], Li et al. [10] reported that the increase observed in total phenolic compounds in Chinese jujube juices after fermentation may also be linked to the activity of hydrolytic enzymes from microorganisms acting on complex phenolic compounds. Furthermore, the results indicated that the used strains have different phenolic acid metabolization capacities. Probiotic strains added with *L. plantarum* recorded higher phenolic acid metabolization rates in blueberry and blackberry juices than those added with *S. thermophilus* and *B. bifidum*. Overall, microorganisms can produce hydrolytic enzymes that in turn act in phenolic complexes. According to the aforementioned authors, species belonging to the genus *Lactobacillus* are capable of producing larger numbers of these enzymes than the ones belonging to other genera.

Probiotic strains, such as *Bifidobacterium lactis* BS 05, *Lactobacillus acidophilus* LA 06 and *Levilactobacillus brevis* LBR01, have already been acknowledged and patented for their antioxidant activity. In addition, there is also evidence in the literature of the antioxidant capacity of *L. plantarum*, *Lactobacillus helveticus*, *L. acidophilus*, *Limosilactobacillus fermentum*, *L. casei*, *Lactobacillus* GG and of some bifidobacterial strains [52]. However, as reported in the herein reviewed articles, antioxidant property is linked to specific strains [10,28].

Microorganisms have increased the antioxidant capacity in 3 of the 5 studies carried out in this analysis, as shown in Table 1. Values may change depending on the used strain. According to Li et al. [10] and Wu et al. [28], probiotics can play a key role in changing the phenolic profile of matrices. The aforementioned authors have also associated this fact with increased antioxidant capacity. However, based on the study conducted by Almeida Bianchini Campos et al. [26] and Valero-cases et al. [35], microorganisms only helped in maintaining high antioxidant capacity. From another perspective, Mustafa et al. [36] have tested the antioxidant capacity in pomegranate juice added with four lactobacillus strains (*Lacticaseibacillus casei* NRRL B-1922; *Lacticaseibacillus casei* NRRL B-227; *Lacticaseibacillus Bulgaria* CFFC B0043; *Ligilactobacillus salivarius* NRRL B-1949) and adjusted the pH to 2.5; 4.0 and 5.5. According to these authors, this parameter was also affected by pH change. The addition of microorganisms has considerably increased antioxidant capacity at pH 4, but this parameter decreased at pH 2.5 and 5.5. According to Mustafa et al. [36], this fact indicates that pH is an important factor to modify the metabolite profiles of fermented juices. The appropriate pH value favors the action of enzymes on the cell wall of the food matrix during fermentation. Thus, it contributes to the release of cell wall components during fermentation and induces the release of phenolic compounds from food matrices, thus contributing to the antioxidant capacity.

Finally, based on the results of the herein reviewed studies, most lactic acid bacteria have oxygen-free radical scavenging systems. Therefore, another possible mechanism to be taken into consideration lies in the synthesis of bioactive peptides as effective antioxidant activity modes in food products added with probiotic bacteria [52].

#### 3.5.4. Instrumental Color Analysis

Color is an important aspect used in food products to attract consumers since it is linked to food taste, nutrition, and quality. Overall, fruit and vegetable juices have pigments that can be altered due to chemical reactions and the microbial growth taking place during fermentation [53].

Studies by De Oliveira et al. [31] and Mokhtari et al. [30] did not report perceptual changes in juice color during storage time, regardless of formulation or the addition of microorganisms. Li et al. [3] have emphasized that Chinese jujube juice fermentation made the samples lighter and less red. On the other hand, Almeida Bianchini Campos et al. [26] observed that fermented pineapple and “juçara” juices showed a higher trend to turn red and yellow, respectively.

Anthocyanins are pigments accounting for the color of several juices; they are easily degraded by chemical reactions under different oxygen, enzyme, pH and temperature conditions [54]. However, these red pigments are more stable in acidic media. Therefore, fermentation and, consequently, the decrease in pH may explain the results reported by Almeida Bianchini Campos et al. [26].

Rengadu et al. [44] assessed color changes in apple juice added with free microorganisms and resistant starch microcapsules. They observed that microcapsules significantly affected juice color. Visual inspection has shown that the control sample acquired a golden yellow color, although the juice turned slightly darker and cloudy after the microcapsules were added to it. According to the aforementioned authors, the observed color variation could result from microcapsules’ dispersion in the juice at the time the samples were analyzed.

Finally, according to Mokhtari et al. [30], although the presence of alginate microcapsules (clear) in contrast to grape juice initially reduced the sample’s color over the storage period, no significant additional change in color was observed. However, changes observed in the product hindered the color sensory analysis. Thus, grape juices added with microcapsules recorded the lowest acceptability rate for this parameter.

#### 3.5.5. Turbidity and Viscosity

Turbidity is an indicator of particle stability and is a decisive visual quality attribute for consumer acceptance of juices. Usually, consumers associate loss of turbidity with deterioration and degradation of quality.

Mokhtari et al. [30] have analyzed turbidity in grape juice added with microorganisms (*Lactobacillus acidophilus*—PTCC 1643 and *Bifidobacterium bifidum*—PTCC 1644), either in their encapsulated or free form, during storage at 4 °C for 60 days. At the beginning of the storage period, turbidity in treatments added with free bacteria was significantly higher than that observed for the control and for samples added with encapsulated bacteria. At the end of the storage period, turbidity in treatments added with free bacteria significantly increased and was slightly higher in juices added with *L. acidophilus*. According to the aforementioned authors, this happened due to the microorganism’s greater resistance and metabolic activity in the medium. Furthermore, a slight increase in turbidity was observed in juices added with microencapsulated microorganisms compared to the control. According to these authors, this happened due to the release of materials from microcapsules, along with the release of metabolites during bacterial growth in the juice. In addition, the contact of calcium alginate in the capsules with minerals in grape juice, mainly with sodium and phosphorus, during the longer storage period, led to gradual capsule structure degradation and, consequently, to increased turbidity.

Garcia et al. [29] assessed the viscosity of apple, orange and grape juices added with five Lactobacillus strains (*Lactiplantibacillus plantarum* 49; *Levilactobacillus brevis* 59; *Lactobacillus paracasei* 108; *Limosilactobacillus fermentum* 111; *Lactiplantibacillus pentosus* 129). The aforementioned authors observed increased viscosity in apple juice added with *L. plantarum* 49, *L. brevis* 59, or *L. fermentum* 111, as well as in the control, for the first time at 21-day storage. However, this parameter decreased in samples with *L. paracasei* 108 or *L. pentosus* 129. Viscosity in orange and grape juices has increased in samples added with microorganisms at 21-day storage, regardless of strain, as well as in control samples. According to these authors, the increased viscosity observed in fruit juices may be associated with the ability of some *Lactobacillus* species to produce exopolysaccharides capable of acting as texturizing agents, as well as to increase the viscosity of the final product and to interact with other juice constituents, such as proteins.

However, since juice samples without lyophilized *Lactobacillus* cells’ addition have also shown increased viscosity, this finding was explained based on the argument that the interaction among compounds found in fruit juices (sugars, pectin, and proteins) can help strengthen hydrogen bonds between solutes and that this process results in a decrease in intermolecular space as well as an increase in the product’s viscosity.

#### 3.5.6. Amino Acids

Only one article assessed the presence of amino acids in probiotic juices. According to the study by Xu et al. [27], 18 amino acids were identified in mixed juice comprising Chinese jujube, apple, orange, and carrot; aspartic acid was the most abundant among them, followed by glutamic acid and proline. The first two acids recorded a post-fermentation decrease, whereas proline recorded a slight increase. The metabolic activity of the investigated three probiotics has changed amino acids’ type and content in the juice; these changes have the potential to change the juice’s flavor during the fermentation process.

#### 3.5.7. Formation of Volatile Compounds

The composition and concentration of volatile compounds are aspects of great interest for the development of fruit and vegetable juices added with probiotics since they have a direct influence on sensory properties as well as product acceptance. Furthermore, only studies conducted by Li et al. [10] and Xu et al. [27] focused on investigating fermentation effects on the profile and content of volatile compounds.

According to Li et al. [10], fermentation significantly improved the formation of volatile compounds, mainly for *L. plantarum* in Muzao Chinese jujube juice and for *L. casei* in Hetian Chinese jujube juice. With respect to the profile of the formed volatile compounds, alcohols were important aromatic compounds found in both fermented juices. They contributed to their light aroma and acted as solvents for other aromatic substances. Still, fermentation had a positive influence on acetaldehyde production to a lesser extent; in other words, it may have given positive aromatic attributes to the investigated juices. Furthermore, acid production increased, mainly in Muzao juice fermented by *L. helveticus* and in Hetian juice fermented by *L. casei*, and it gave a sour taste to the juice. According to the aforementioned authors, ketones and esters always give a pleasant odor to food products. Fermentation increased ketone formation, mainly in Muzao juice fermented by *L. plantarum* and in Hetian juice fermented by *L. casei*. Samples fermented by *L. plantarum* also showed an increase in esters content. Based on these results, these authors have emphasized that aroma development in fermented juices is a complex and dynamic process since different strains have different metabolic patterns in different environments.

On the other hand, Xu et al. [27] identified 36 compounds in mixed juices comprising Chinese jujube with orange, carrot, and control apple, whereas 34 compounds were found in the fermented juice. Alcohols and alkenes were the total volatile compound classes prevailing in the analyzed samples.

The aforementioned authors also identified a few aldehydes in the analyzed samples, which can be attributed to their instability in food matrices. Therefore, they were likely reduced to alcohol or oxidized into acids due to microbial activity. This is a positive result, since, according to these authors, high aldehyde concentrations lead to a reduction in product acceptability. Finally, total ester content in the fermented juices significantly increased. Esters are widely studied and appear to contribute to fruity notes. Yet, the aforementioned authors declared that the fermented juices presented intense floral and fruity notes that in turn may be associated with their high alcohol, ketone, and terpene contents.

In short, both previously mentioned studies included an analysis of volatile compounds in their investigation process. Despite being a complex process, the addition of probiotic microorganisms and, consequently, the fermentation process have a positive impact on the formation of volatile compounds. It is essential to point out that this finding may be associated with the adopted conditions since both studies used Chinese jujube juice as the matrix, even at different ratios and combinations, as well as the addition of *L. plantarum* strains for fermentation purposes.

#### 3.5.8. Microbiological Analysis

Microbiological analysis is essential to help collect information about contaminating microorganisms and, consequently, to guarantee the safety of the product to be developed [55]. However, only 4 of the 21 articles included in the current review performed this analysis.

Molds and yeasts are the most common contaminating microorganisms found in fruit and vegetable juices since they can multiply under these products’ acidic conditions. On the other hand, bacterial growth in fruit juice depends on pH, humidity, temperature, and storage time, as well as on water activity, preservative concentration, treatment application, sugar content and the amount of raw material [55].

Accordingly, all herein assessed studies performed a mold and yeast analysis, except for the study by Almeida Bianchini Campos et al. [26]. Valero-cases and Frutos [32] subjected carrot and orange juices to pasteurization (90 °C/5 min), as well as to microbiological analysis of molds and yeasts, during 30-day storage at 4 °C. The fermented juice did not show colony-forming units, whereas non-fermented juices presented numbers of molds and yeasts lower than 3 log_10_ CFU/mL. Based on this result, the aforementioned authors justified that, in addition to thermal treatment, the fermentation of juices by probiotic microorganisms can help maintain their microbiological safety and prolong their shelf life since this process can inhibit contaminating flora growth by increasing lactic acid levels and decreasing pH in juices.

From another perspective, Olivares et al. [38] and Bonaccorso et al. [43] compared the microbiological features of juices added with free or microencapsulated microorganisms. However, the results in their study were divergent.

Olivares et al. [38] analyzed the growth of aerobic microorganisms, molds, and yeasts, as well as *E. coli* in pasteurized pineapple, raspberry, and orange juices (88 °C/90 s) stored under refrigeration (4 °C) for 28 days. All juices added with free bacteria, except for the pineapple juice, presented numbers of molds, yeasts and aerobic bacteria lower than those observed for juices added with microencapsulated microorganisms. *E. coli* results were negative in all samples. Probiotic microorganisms in free forms may have directly affected the survival of contaminating microorganisms. These results may be related to the composition, pH value, presence of antimicrobial compounds and competition for space and nutrients in the juices.

On the other hand, Bonaccorso et al. [43] observed the multiplication of microorganisms such as *Leuconostoc* spp., mesophiles, psychotrophics, molds and yeasts in orange juice free from prior treatment or the addition of preservatives. Furthermore, the authors observed that the number of microorganisms was higher in samples added with free probiotic bacteria than in those added with microencapsulated microorganisms.

Although these authors did not present a justification for these results, they mentioned that microencapsulation could be a promising approach to help preserve juices since it improves their stability. Although fruit juice is a suitable matrix used to grow spoilage microorganisms, such as molds and yeasts, and *Leuconostoc* spp., this strategy was more effective in reducing the excessive growth of microorganisms in comparison to the addition of free probiotics.

It is worth mentioning that there were studies carried out in different countries among the herein selected articles; thus, each article has followed a specific legislation to check the safety of the developed juices. Almeida Bianchini Campos [26], for example, investigated *E. coli* and *Salmonella* sp. in mixed pasteurized pineapple and jussara juice (88 °C/2 min). The aforementioned authors reported *E. coli* values lower than 1 log_10_ CFU/mL and a lack of *Salmonella* sp. in 25 mL of sample. These findings met the requirements of the current Brazilian legislation. Bonaccorso et al. [43] stated that values recorded for contaminating microorganisms in all samples were in compliance with the safety limits set by the European Union legislation. Finally, Olivares et al. [38] used the Chilean legislation based on the *Codex Alimentarius* as a reference to classify juice samples as safe.

#### 3.5.9. Sensory Analysis

The effects of probiotics on the sensory features of juices depend on fruit type, probiotic organism, storage temperature, and supplementation with prebiotics and protectants. However, there is an increase in acidity levels as well as a decrease in sweetness levels during the fermentation or storage period of probiotic fruit juices. This happens due to sugar consumption for the growth of microbial cells or maintenance purposes; this process can lead to undesirable changes in the flavor of the juices as well as decrease their acceptability [35,52,55].

Accordingly, all studies performing this analysis recorded sensory changes as well as lower scores on attributes and acceptability rates after fermentation, except for the study conducted by Wu et al. [28], who conducted a sensory analysis of blackberry and blueberry juices fermented by three probiotic bacteria strains (*Lactiplantibacillus plantarum* BNCC 337796; *Streptococcus thermophilus* CGMCC1.8748; *Bifidobacterium bifidum* CGMCC1.5090). The aforementioned authors observed that blueberry juice added with *L. plantarum* recorded the highest scores in the overall sensory assessment in comparison to all other samples. However, the opposite was observed for blackberry juice. Thus, a likely solution for juices whose overall acceptance may be negatively affected by probiotics lies in using strategies to mitigate this impact from a sensory perspective, i.e., the addition of ingredients such as volatile compounds or even other fruit juices.

Thus, studies such as the ones conducted by Dimitrovski et al. [39] and Güney and Güngörmüşler [40] have added juices made of other fruits to help mitigate the impacts of probiotic microorganisms on the investigated products’ sensory features. These authors observed that the initial juice acceptance increased when this approach was adopted to prepare juices [23,52,56]. Therefore, probiotic microorganisms can negatively affect the sensory quality of juices depending on the used matrix. Thus, the proper selection of fruits and vegetables can be a decisive factor in the acceptance of the prepared probiotic product.

### 3.6. Microorganisms’ (Microencapsulated x Free) Inoculation Method and Changes in Juice Features and in Microorganisms’ Viability

Studies focusing on comparing physical-chemical parameters between products inoculated with free and microencapsulated probiotics observed the most intense changes in juices added with free bacteria. The main argument used to explain this finding was that free bacteria would have unrestricted access to nutrients in the juice and that it would consume more sugars. Consequently, it would lead to higher acidity as well as decreased pH and total soluble solids [30,38,43,44,45].

On the other hand, all articles focused on comparing viability have observed a larger number of viable cells in juices added with microencapsulated probiotics. The explanation given by most authors for this outcome was that these microcapsules could work as a physical barrier to protect microorganisms from adverse environmental conditions, such as low pH [30,38,43,44].

Mokhtari et al. [30] had the only study that focused on assessing sensory features among the herein selected ones. According to the authors, encapsulation can change the perception about the color and appearance of fruit juices. Moreover, microcapsules in juices likely change the product’s mouthfeel, mainly because they are liquid products. On the other hand, a higher overall acceptance level was observed for products added with microencapsulated bacteria. Thus, encapsulation can enable a more controlled environment and therefore lead to fewer changes in the product’s taste [30].

## 4. Final Considerations

Based on the herein analyzed studies, it was found that, overall, fruit and/or vegetable juices can be propitious matrices used to develop probiotic foods. However, above all, the added microorganisms must be able to adapt to and survive in the environment to guarantee the product’s success.

The results of the current study have shown that pH is one of the most critical factors enabling the survival of microorganisms. In addition, the synergy between the food matrix and added strain, as well as fiber content, phenolic compounds, and amino acids, also plays an essential role in the maintenance of probiotic microorganisms inoculated in juices. Furthermore, microencapsulation is a promising technology to help improve the viability of microorganisms by creating a more controlled environment with fewer physicochemical and, likely, sensory changes.

On the other hand, fermentation can lead to undesirable sensory changes, which can hinder the process of developing juices with probiotic fruits and vegetables. However, fermentation can help improve the product’s microbiological safety since it appears to inhibit the growth of contaminating microorganisms.

It is also important to emphasize that probiotic microorganisms play an essential role in improving the antioxidant activity of the matrices they are inoculated in. However, this effect depends on the added strain.

Finally, given the wide variety of analyses conducted in the herein assessed studies, the main limitation of the present study refers to the comparison between parameters. Therefore, future studies should focus on filling the remaining gaps observed in the development of probiotic fruit and/or vegetable juices and mixed juices to develop a consensus about this process.

## Figures and Tables

**Table 1:** Studies focused on assessing the viability and impacts of adding probiotic microorganisms to fruit and mixed juices: Main analyses carried out, juice type, inoculated microorganism, study conditions, and main results.

Juice Type	Main Analyses Carried Out	Inoculated Microorganism	Study Conditions	Main Results	Reference
Litter	-Viability -pH	*Bacillus coagulans MTCC 5856* Inoculum: 10^7^ Addition: free form.	Six months. Under refrigeration (4–6 °C).	**pH:** No changes in initial pH were observed up to 6 months. **Viability:** *B. coagulans* MTCC 5856 stable with viability higher than 99%.	[33]
Pomegranate/ orange/carrot	-Viability -pH -Total titratable acidity -Total sugars	*Weissella kimchii* R-3 Inoculum: ~10^9^ Addition: free form.	Fermentation (37 °C/72 h). Storage (5 weeks—4°C/12 days—25 °C).	***Fermentation:*** **Viability:** Viability decreased in pomegranate and orange juices but increased in carrot juice within 24 h. After 48 h, viability in carrot juice kept on increasing, but it remained constant in pomegranate and orange juices. **pH and acidity:** pH decreased in all juices in the first 24 h. The acidity of pomegranate and orange juices remained virtually constant, although it increased in carrot juice. **Total sugars:** Reduced in all juices during the first 24 h (42% pomegranate juice, 22.7% orange juice and 9% carrot juice). Sugar content in carrot juice decreased by 50% at 48–72 h. ***Storage:*** **Viability:** Viability in pomegranate and orange juices gradually decreased to zero within two weeks. Carrot juice showed better viability under both storage conditions. **pH and acidity:** pH slightly decreased in pomegranate and orange juices towards the end of the storage period. A slightly sharper decrease was observed in carrot juice. Acidity levels only considerably increased in carrot and orange juices during storage at room temperature. Acidity only increased in pomegranate juice and decreased in orange and carrot juices at the end of 5-week refrigerated storage. **Total sugars:** All juices presented a decrease in total sugar content (there was a significant decrease in total sugar content in orange and carrot juices), both in refrigerated storage and in storage at room temperature. The most significant decrease in sugar content in pomegranate juice was observed at room temperature, whereas the opposite was observed for carrot and orange juices.	[34]
Carrot and orange	-Viability -pH -Organic acids -Total titratable acidity -Total Sugars -Microbiological analysis -Inulin	*Lactiplantibacillus plantarum* subsp. *plantarum* CECT 220 Inoculum: 10^8^ Addition: free form.	Fermentation (37 °C/24 h). Storage (30 days/4 °C).	**Viability, total sugars, pH, and organic acids:** After fermentation, probiotic viability was approximately 10^9^ CFU/mL in all juices added with the microorganism, and it remained close to this value during storage time. Increased lactic acid level associated with decreased fructose, glucose, and malic acid levels was observed; there was no significant difference between juices. In addition, pH decreased from 4.9 to 3.9 in all fermented juices. Citric acid levels in all juices remained unchanged during fermentation and storage times. **Microbiological analysis of molds and yeasts:** They were not detected in any of the fermented juices during the test. However, the unfermented control juice showed concentrations of these elements higher than >10^3^ CFU/mL after refrigerated storage for 15 days.	[32]
Pomegranate	-Viability -Phenolic compounds -Antioxidant capacity	*Lactobacillus acidophilus* CECT 903 (LA), *Lactiplantibacillus plantarum* subsp. *plantarum* CECT 220 (LP), *Bifidobacterium longum* subsp. *infantile* CECT 4551 (BL), *Bifidobacterium bifidum* CECT 870 (BB). Inoculum: 10^6^ Addition: free form.	Fermentation (24 h/4 °C and 37 °C)	**Viability:** Strains increased from 10^6^ CFU/mL to 7.26–7.78 Log_10_ CFU/mL, although there was no significant difference between the used bacteria. **Phenolic compounds:** Eight phenolic compounds were found in pomegranate juice (catechin, α and β punicalagin, punicalin, epicatechin, gallic acid, ellagic acid derivative, and ellagic acid). Fermented samples have shown increased phenolic compound levels as well as a new catechin derivative. Overall, *B. longus* subsp. was the strain with the least impact on phenolic compound contents. β-punicalagin and α-punicalagin concentrations in pomegranate juices fermented by *Lactobacillus* were lower than those observed in juices fermented by *Bifidobacterium* strains. **Antioxidant capacity:** Fermentation increased the antioxidant capacity.	[35]
Pineapple and jussara juice	-Viability -pH and Total Titratable Acidity -Total soluble solids -Total phenolic compounds -Antioxidant capacity -Instrumental color analysis -Microbiological analysis Anthocyanins	*Lacticaseibacillus rhamnosus* GG Inoculum: 10^10^ Addition: free form.	Fermentation (24 h/37 °C) Storage (28 days/8 °C)	**Viability:** Microorganism counts were higher than 7.2 log_10_ CFU/mL over 28-day storage. **pH and Total Titratable Acidity:** Juice fermentation reduced pH value and increased titratable acidity between 0 and 3 days. pH and acidity values remained stable during storage time. **Total soluble solids:** No differences in total soluble solids were observed between treatments or at 28-day storage time. **Total phenolic compounds:** Control juice and fermented juices differed from each other in total phenolic compound contents, although storage time was insignificant in this parameter between these same treatments. **Antioxidant capacity:** There was no difference in antioxidant capacity between treatments or throughout the storage period. **Instrumental color analysis:** There were no differences in lightness (L*) between treatments. On the other hand, coordinates a* (greater tendency towards red—a more stable form of anthocyanins) and b*(greater tendency towards yellow) differed between the control juice and the one added with probiotic bacteria. **Microbiological analysis:** *E.coli* count was lower than 1 log_10_ CFU/mL; no sample presented *Salmonella* sp.	[26]
Apple, orange, and grape	-Viability -pH -Total titratable acidity -Organic acids -Total sugars -Total soluble solids -Instrumental color analysis -Viscosity	*Lactiplantibacillus plantarum* subsp. *plantarum* 49; *Levilactobacillus brevis* 59; *Lacticaseibacillus paracasei* 108; *Limosilactobacillus fermentum* 111; *Lactiplantibacillus pentosus* 129 Inoculum: 10^8^–10^9^ Addition: free form.	Storage (21 days/4°C)	**Viability:** All strains decreased in all fruit juices over the storage period. *L. paracasei* 108 and *L. fermentum* 111 recorded the highest and lowest survival rates in juices over 21 days, respectively. **pH and Total Titratable Acidity:** These parameters did not change until the 14th day; pH in apple juice and grape juice added with *L. brevis* 59, *L. paracasei 108*, *L. fermentum* 111, or *L. pentosus* 129 increased at the 21st day in comparison to the 1st day. pH in grape juice added with *L. plantarum* 49 and in orange juice (intensity changed depending on the strain) decreased at the 21st day. Conversely, titratable acidity in apple juice added with *L. fermentum* 111 or *L. pentosus* 129, as well as in orange and grape juice added with *L. plantarum* 49 *or L. brevis* 59, increased on the 21st day. **Total soluble solids:** Values increased in apple juice added with *L. brevis 59*, *L. fermentum* 111, or *L. pentosus* 129, as well as decreased in orange juice, regardless of strain or microorganism addition, on the 21st day of storage. No change in values was observed in grape juice samples regardless of strain or microorganism addition (or not). **Color:** Changes in L*, a*, or b* values for fruit juice over time were verified, regardless of *Lactobacillus* cells addition to it. L* value (luminosity) decreased in apple and grape juices and increased in orange juice during storage. The a* value increased in apple and grape juices, but it did not change in orange juice. The b* value did not change in apple juice, but it decreased in grape juice and increased in orange juice over time. Significant modification in color change was verified in all juices at the 21st day of storage, except for apple juice added with *L. paracasei* 108 or *L. fermentum* 111. **Organic acids and total sugar content:** The highest malic, citric, and tartaric acid levels were observed in apple, orange, and grape juices without *Lactobacillus cells* addition, respectively, and did not change over storage time. Malic and lactic acid contents decreased and increased overtime in juice samples added with *L. paracasei 108* and *L. plantarum 49*, respectively. Succinic acid was only detected in orange juice added with *L. paracasei 108* or *L. plantarum 49*. Tartaric acid content decreased in grape juice added with *L*. paracasei 108 or L. plantarum 49 during storage time. Tartaric acid content did not change overtime in apple juice added with *Lactobacillus* cells. Citric acid content decreased over-time in apple and grape juices added with *L*. *paracasei 108* or *L. plantarum 49*, respectively. Citric acid content did not change in fermented orange juice.	[29]
Grape	-Viability -pH -Total titratable acidity -Instrumental color analysis -Sensory analysis -Turbidity	*Lactobacillus acidophilus* (PTCC 1643) *Bifidobacterium bifidum* (PTCC 1644) Inoculum: 10^9^–10^10^ Addition: free form and microencapsulated	Storage (8 weeks/4 °C)	**Viability during storage:** With respect to both strains assessed in the study, the final population (day 60) of encapsulated bacteria was significantly higher. Comparison between strains has evidenced that *B. bifidum* showed a more intense (non-significant) decline than *L. acidophilus.* **pH:** This decreased in all juice formulations during storage time. Juices added with free bacteria showed a sharper pH reduction; the highest values were observed in juices added with *L. acidophilus*. Samples added with encapsulated bacteria did not show a significant difference in pH between *L. acidophilus* and *B. bifidum.* **Acidity:** It increased in all treatments (except for the control) for 60 days; the highest value was recorded for treatments added with free bacteria. **Instrumental color analysis:** The color of all samples was different from that of the control at the beginning of the storage time. Color variation in samples added with encapsulated bacteria was more significant than that of samples added with free bacteria. Changes in parameter * L (luminosity) were observed in juices added with free bacteria due to higher medium turbidity. However, the color of encapsulated treatments did not change until the 60th day of storage. Bacterial activity in treatments added with free probiotics also did not significantly affect juice color. **Sensory analysis:** Samples added with encapsulated microorganisms recorded low color scores during storage time. However, bacterial type and sampling day did not affect the results. In addition, encapsulation treatments recorded lower scores for mouthfeel. The control group presented better overall acceptance in the last 60 days; this was followed by groups added with encapsulated and free *B.bifidum*, as well as by groups added with encapsulated and free *L. acidophilus*. However, the taste of *L. acidophilus*-free samples was reported as undesirable due to acidity in this treatment.	[30]
Pomegranate	-pH -Phenolic compounds -Viability -Antioxidant capacity	*Lacticaseibacillus casei* NRRL B-1922; *Lacticaseibacillus casei* NRRL B-227; *Lactobacillus delbrueckii subsp. bulgaricus* CFFC B0043 *Ligilactobacillus salivarius* NRRL B-1949 Inoculum: ~10^9^–10^10^ Addition: free form	Different temperatures (30 °C, 35 °C, 37 °C/24 h) pH adjustment (2.5; 4.0; 5.5)	**pH:** The results showed a slight drop in pH—from 3.58 to 3.17—during fermentation time. **Phenolic compounds:** The following compounds were found in fermented pomegranate juices: phenolic acids (rosmarinic and citric acids) and flavonoids (quercetin, quercetin-3-glucoside, rutin and kaempferol rutinoside). The total phenolic content in these juices decreased after 24-h fermentation, but 70% of its content was maintained at the highest temperature (37 °C,) in comparison to approximately 60% of it, at 30 °C and 35 °C. There was a significant decrease in phenolic content at different pH values, mainly at pH 5.5. Thus, total phenolic compounds appear to be more affected by pH adjustment than by temperature. **Viability:** All bacterial strains grew well in pomegranate juice (increased biomass). *L. casei* showed the highest biomass, mainly at 35 °C and 37 °C, and it was selected to be used in the following tests. Bacterial growth suppression was observed at pH 2.5 and 5.5 (it was more significant at 5.5). Adjusted pH values below or beyond the initial pH (3.58) were capable of suppressing *L. casei* growth. Cell viability increased more than three times its initial value at pH 4.0. **Antioxidant capacity:** Fermentation with *L. casei* increased juice’s antioxidant capacity at pH 4.00. However, adjusted pH of 2.5 and 5.5 led to significantly reduced antioxidant activity. Fermentation with probiotic bacteria could contribute to maintain high antioxidant capacity.	[36]
Cranberry/ lemon and Tahiti/ pomegranate	-Viability during storage in cells previously dapted, or not, to different pH values.	*Lacticaseibacillus plantarum* NCIMB 8826 Inoculum: ~10^8^ Addition: free form	Storage (3 days for cranberry juice; and six weeks for lemon and pomegranate juices)	**Cranberry juice:** The viability of cells previously adapted to pH 3 significantly improved in comparison to that of non-adapted cells. Cells adjusted in acidified MRS pH 3 and 4 were capable of surviving in cranberry juice for 72 h at concentrations of 10^3^ CFU/mL and 10^2^ CFU/mL, respectively. **Lime and Sicilian lemon juice, and pomegranate juice:** Cell survival rate in Lime and Sicilian juice as well as in pomegranate juice was higher than that observed for cranberry juice. Cells adapted to MRS acidification (pH 3) in these juices presented 1 log CFU/mL more than control cells during the 1st and 2nd storage weeks. However, significant differences were not observed from the 3rd week onwards.	[37]
Raspberry/ pineapple/ orange	-Viability -Microbiological analysis	*Lacticaseibacillus casei* (DSM 20011) Inoculum: ~10^7^ Addition: free form	Storage (28 days/4 °C)	**Viability:** **Pineapple juice:** Some microcapsules did not resist the acidity of this juice; *Lactobacillus* found in them were released into it, a fact that increased the count of these microorganisms. On the other hand, more than 65% of microcapsules were recovered with 2.3 × 10^7^ CFU/g (the same value as the initial microcapsules) after 28 days. Thus, there was no loss of viability in microcapsules. With respect to pineapple juice added with free microorganisms, the count remained almost constant during the storage period (viability higher than 95%) throughout the storage time. **Orange juice:** The *Lactobacillus* count increased in orange juice after the first storage week, with the number of viable cells reaching 7.0 × 10^4^ CFU/mL. After 28 days, 59.3% of microcapsules were recovered with 5.5 × 10^6^ CFU/g, which represented 91% of the initial viability. Viability significantly decreased in juice added with free microorganisms after 14-day storage, whereas lactobacillus count was virtually zero on the 21st day. However, viability reached 10^3^ CFU/mL at the end of storage time. **Raspberry juice:** Probiotic bacteria were released from microcapsules into the medium on the 7th day, and their count slightly increased towards the end of storage time (>2.2 × 10^5^); 47.6% of microcapsules were recovered. Juices added with free microorganisms presented a remarkable loss of viability on the 7th day and a total lack of cells on the 14th day. **Microbiological analysis:** *E. coli* tests recorded negative results for all three juices, and the number of aerobic microorganisms was in compliance with the Chilean sanitary legislation, based on the *Codex Alimentarius*.	[38]
Mixed juice of Chinese jujube, apple, orange, and carrot	-Viability -pH -Total titratable acidity -Volatile compounds -Reducing sugars -Aminoacids -Organic acids	*Lactiplantibacillus plantarum* CICC20265 *Bifidobacterium breve* CICC6184 *Streptococcus thermophilus* CICC6220 Inoculum: 10^7^ Addition: free form	Fermentation and Storage (3 weeks/4 °C)	**Viability during fermentation and storage:** Viable cell count at the end of fermentation reached 4.36 × 10^8^ CFU/mL; it was 7.56 × 10^8^ CFU/mL at the end of the storage time. **Reducing sugars:** They significantly decreased after fermentation. **pH:** A pH of 3.29 was observed at the end of fermentation as well as a pH of 2.80 at the end of the storage time. **Organic acids and acidity (lactic acid):** Malic, citric, and tartaric acid contents significantly decreased after fermentation. However, lactic acid content substantially increased throughout fermentation.	[27]
Pumpkin	-Viability -pH -Total titratable acidity -Total sugars -Sensory analysis	*Lacticaseibacillus casei* 431 Inoculum: 10^7^ Addition: free form	**Fermentation** (37 °C/48 h) Storage (10 days/4–7 °C)	***Fermentation:*** **Viability:** Cell count increased from 10^6^ to 10^10^ log CFU/mL in 24 h; it remained constant until the end of the fermentation time (48 h). **pH and acidity:** There were no changes in pH or acidity levels in the first 5 h. Afterwards, pH progressively decreased until it reached 3.6, in 33-h fermentation. After this period, there was no significant change in it until the end of the fermentation time. For acidity, an increase was found. **Total sugars:** Glucose was the primary source of carbon and energy used by *L. casei* 431; a small fructose fraction was also used, and sucrose was the most abundant sugar in the juice. ***Storage:*** **Viability:** Viability remained close to 10^6^ CFU/mL after 10 days. **Sensory analysis:** Mixed juices were prepared to increase pumpkin juice’s acceptability (pumpkin juice + apple juice; pumpkin juice + blueberry juice; kiwi and apple juice; pumpkin juice + orange, carrot and lemon juice) in the sensory analysis. Pumpkin juice and blueberry juice scored the highest values, whereas pure fermented pumpkin juice scored the lowest values for all sensory perceptions, except for color. There was no significant difference between samples for smell and color attributes.	[39]
Two types of Jujube (*Ziziphus Jujuba* cv. Muzao and Hetian)	-Viability -Total soluble solids -pH -Total sugars and reducing sugars -Total titratable acidity -Organic Acids -Phenolic compounds -Antioxidant capacity -Volatile compounds -Instrumental color analysis	*Lactobacillus acidophilus* 85; *Lacticaseibacillus casei* 37; *Lactobacillus helveticus* 76; *Lactiplantibacillus plantarum* 90 Inoculum: ~10^8^ Addition: free form.	Fermentation (37 °C/48 h)	**Viability:** There was no significant difference in the growth capacity of strains in both juices, which recorded values higher than 10^11^ CFU/mL at the end of fermentation. **Total soluble solids:** A slight drop from 10°Brix to 9.5°Brix. **pH:** A significant reduction from 5.00 to 3.74–3.82. **Total sugars and reducing sugars:** Both juices presented considerably decreased total and reducing sugar levels during the fermentation time. *L. acidophilus* had a stronger impact on the total sugar content in Muzao juice, whereas L. *plantarum* had a stronger impact on this parameter in Hetian juice. **Total titratable acidity:** Acidity significantly increased at the end of the fermentation time. The highest titratable acidity value was observed in Muzao juice fermented by *L. helveticus* and in Hetian juice fermented by *L. casei*. **Organic acids:** Tartaric and lactic acids prevailed in Muzao juice, whereas tartaric and malic acids prevailed in Hetian juice. Lactic acid content significantly increased after fermentation. On the other hand, tartaric and citric acid contents also decreased after fermentation. **Phenolic compounds:** Total phenolic content and total flavonoids increased and decreased after fermentation, respectively. Fermentation had a significant impact on the phenolic profile of the jujube juices. Protocatechuic acid, caffeic acid, and rutin contents increased after fermentation in Muzao juice fermented by *L. plantarum*. The contributions of different strains to phenolic profiles differed in Hetian juice: gallic acid content increased after fermentation by *L. plantarum*, rutin content increased after fermentation by *L. casei,* epicatechin and cinnamic acid content increased after fermentation by *L. acidophilus*, and caffeic acid content increased after fermentation by *L. helveticus*. **Antioxidant capacity:** Antioxidant capacity improved after the addition of microorganisms to both juices. **Volatile compounds:** A total of 74 volatile compounds were identified and quantified in jujube juices. Fermentation significantly improved the formation of volatile compounds; consequently, it improved the aroma of the analyzed juices, mainly of Muzao jujube juice fermented by *L. plantarum* and Hetian jujube juice fermented by *L. casei*. **Colorimetric analysis:** A* decreased, and L* increased in Muzao and Hetian juices after fermentation. This outcome means that the addition of microorganisms made jujube juices lighter and less red. Still, fermented Hetian juices recorded increased *b, and it indicated juice yellowing; the opposite was observed for Muzao jujube juices (except for the one fermented by *L. acidophilus)*. The fermentation of juices also increased their overall color difference from the control, mainly for Hetian juices. The smallest difference was observed for Muzao juice fermented by *L. acidophilus.*	[10]
Jerusalem artichoke, pineapple, pumpkin, spinach, and Cucumber	- Total soluble solids -Viability -pH -Organic acids -Sensory analysis	*Lacticaseibacillus rhamnosus* ATCC 53103; *Lacticaseibacillus paracasei* subsp. *paracasei* ATCC55544; *Lactobacillus acidophilus* La-5 DSM 15954; *Lactiplantibacillus plantarum* DSMZ 20174; *Bifidobacterium animalis* subspecies *lactis* BB-12 DSM 15954 Inoculum: 10^9^ Addition: free form.	**Fermentation** (24h/37 °C) **Storage** (45 days/8 °C)	**Total soluble solids:** There was a drop in °Brix values ranging from 0.7% to 2.3% after fermentation. Juice added with *L. plantarum DSM 20174* recorded the highest Brix value. On the other hand, the lowest value was observed for the juice added with *L. rhamnosus ATCC 53103*, whose Brix value decreased by 1.3% on average. **Viability:** All investigated microorganisms maintained the minimum number of viable cells necessary to exert probiotic activity after 45 days. However, the highest viable count was observed for the juice added with *L. paracasei* subsp. Paramarried ATCC 55544 (9.42 log_10_ CFU/mL) at the end of the fermentation time. However, the juice added with *L. rhamnosus* ATCC 53103 has maintained the highest viability value (9.3 log_10_ CFU/mL) at the end of the storage time. **pH and organic acids:** The pH of all juices decreased during fermentation and storage time. The lowest pH value was observed in the juice added with *L. plantarum* DSM 20174 (pH = 3.02), during storage time. However, the sharpest decrease was recorded for juices added with *L. paracasei subsp. paracasei* ATCC 55544 and *B. animalis subsp. lactis. L. plantarum* DSM 20174 demonstrated the highest lactic acid production capacity among the tested matrices. Furthermore, the highest acetic acid: lactic acid ratio was observed for *L. plantarum DSM 20174,* whereas the lowest one was observed for *L. rhamnosus ATCC 5310* at the end of the fermentation time. **Sensory analysis:** It was applied to samples added, or not, with apple juice. Juice sweetness remained low and acceptance scored 4 out of 7 points. Samples added with apple juice recorded the highest score for overall acceptability. Aftertaste, sweetness, and purchase intent significantly differed between samples, indicating the panelists’ preference for flavor-boosted juices.	[40]
Carnelian cherry (*Cornus mas*)	-pH -Total titratable acidity -Reducing carbohydrates -Total soluble solids -Viability -Sensory analysis	*Lactobacillus delbrueckii* DSMZ 15996; *Lactobacillus acidophilus* 946744 Inoculum: 10^9^ Addition: free form	**Fermentation** (72 h/30 °C) **Storage** (4 weeks at refrigerated temperature)	***Fermentation:*** **pH and acidity:** There were no differences in pH and acidity values recorded for the analyzed samples between 0 and 24 h. The lowest pH and the highest acidity values were observed for juice added with *L. delbrueckii* at 48 h. Juice added with *L. delbrueckii* also recorded the highest acidity value, whereas the control group recorded the lowest pH value at 72 h. Yet, there was no significant difference between juices added with microorganisms. The control remained stable throughout the fermentation time. **Reducing carbohydrates:** The lowest reducing carbohydrate content was recorded for juices added with *L. acidophilus* at 24 h. However, the lowest reducing carbohydrate content was found in samples added with *L. delbrueckii* between 48 and 72 h. The control group recorded the highest values for this parameter throughout the fermentation time. **Total soluble solids:** There was only a difference between strains in total soluble solids in the 48-h interval, when the lowest value was observed for juice added with *L. delbrueckii.* The control group recorded the highest total soluble solids values throughout the fermentation time. ***Storage*** **Viability:** Viable cell count in cornelian cherry juice added with *L. delbrueckii* was significantly higher than that of other treatments. The bacterial population significantly decreased overtime, reaching zero in the 3rd week of storage in juice added with *L. acidophilus,* as well as 7.41 log_10_ CFU/mL in the 4th week of storage in juice added with *L. delbrueckii.* **Sensory analysis:** *L.acidophilus* treatment odor and taste was significantly more acceptable than those of the *L. delbrueckii* treatment and the control group after 4 weeks. However, no difference in color between treatments was observed.	[41]
Mango and carrot	-pH -Total titratable acidity -Total soluble solids and color -Viability -Colorimetric analysis -Total fiber content -Sensory analysis	*Lactiplantibacillus plantarum* LP 299V *Lacticaseibacillus rhamnosus* GG *Lactobacillus acidophilus* LA–14 Inoculum: ~10^10^ Addition: free form	**Fermentation** (24 h/37 °C) **Storage** (35 days/8 °C)	**pH and acidity:** There were a decrease in pH as well as an increase in acidity level in all probiotic mixed juices during storage time. **Total soluble solids and color:** No difference was observed for products’ total soluble solids and color. **Viability:** There was no significant reduction in the count of microorganisms evaluated during storage time, regardless of the adopted formulation. *L. plantarum* and *L. acidophilus* were the microorganisms recording the highest and lowest viability values, respectively. **Sensory analysis:** Juices with higher mango pulp concentration were the most accepted ones.	[31]
Apple/ Orange/ Tomato	-pH -Total Titratable acidity -Viability	*Fructilactobacillus sanfranciscensis* Inoculum: ~10^8^ Addition: free form	**Storage** (4 weeks/4 °C)	**pH:** All juices demonstrated a significant pH reduction after 4-week storage. **Total Titratable Acidity:** Orange and tomato juice acidity increased as pH increased. **Viability:** It significantly decreased in all juices at the end of the 4-week storage (0.52, 0.18 and 0.53 log cfu/mL for apple, orange and tomato juices, respectively). All three juice samples reached the recommended viability level (>10^6^ CFU/mL) for probiotic food types after 4-week storage.	[42]
Orange	-Viability -pH -Total Soluble Solids -Microbiological analysis	*Lacticaseibacillus rhamnosus* Inoculum: ~10^9^ Addition: free form and microencapsulated	**Storage** (35 days/5 °C)	**Viability:** The number of viable cells in samples added with encapsulated microorganisms ranged from approximately 9.0 log_10_ CFU/mL at baseline to 8.3 log_10_ CFU/mL after 35-day refrigerated storage. On the other hand, juice added with free microorganisms presented a lower number of viable cells. (7.93 log_10_ CFU/mL at the beginning; and 5.58 log_10_ CFU/mL at the 35th day). **pH: Overall**, pH value remained almost constant both in treated and untreated juices. **Total Soluble Solids:** No significant change in the value of soluble solids was observed for any sample tested during storage time. **Microbiological analysis:** Microorganism counts recorded for the control group were lower than those recorded for orange juice samples added with encapsulated microorganisms. The addition of free microorganisms to the samples further favored an increase in other microbial groups. There were no visual differences between the tested juices.	[43]
Litter	-Viability -pH -Instrumental color analysis	*Lacticaseibacillus casei* ATCC 334; *Bifidobacterium animalis* ATCC 25527 Inoculum: ~10^10^ Addition: free form and microencapsulated	**Storage** (28 days/4 °C)	**Viability:** Initial free bacteria viability was approximately 10 log_10_ CFU/mL and it dropped to approximately 3 log CFU/mL in microorganism-added juices after 28 days. Bacteria were released from microcapsules on the 7th day; counts ranged from 2 to 3 log_10_ CFU/mL, and there was an increase from 3.2 to 3.8 log_10_ CFU/mL. The viability of the bacteria that remained in the microcapsules was approximately 7 log_10_ CFU/mL. Microencapsulated bacteria recorded viability and stability values higher than those observed for the ones added in their free form. **pH:** It decreased in juices added with free cells and in juice added with microencapsulated cells. **Instrumental color analysis:** The addition of resistant starch microcapsules to the samples had a significant impact on juice color. Control samples turned golden yellow during visual inspection; however, the juice became slightly darker and cloudy after microcapsules were added to it.	[44]
Orange Apple	-Viability during refrigerated storage -pH -Total soluble solids	*Lactobacillus acidophilus* LA-02 Inoculum: ~10^10^ Addition: free form and microencapsulated		**Viability:** Overall, the viability of microorganisms during storage time was higher in orange juice. With respect to the free or microencapsulated addition form, *L. acidophilus* showed greater viability when it was added to fruit juices in the microencapsulated form. Cross-linking was essential to prolong viability (the highest viability until the end of the storage period—8.12 log _10_ CFU/mL) over the storage time. There was no association between increased probiotics concentration and increased viability. **pH:** pH reduction was sharper in orange juice added with microorganisms, and it indicated no metabolic inactivation of probiotics. On the other hand, smaller pH variations were observed in apple juices. Moreover, the highest pH variations took place in treatments added with free cells. **Total soluble solids:** It decreased in samples added with free cells at 10% and 30% concentrations in both fruit juices. Juices added with encapsulated microorganisms at a concentration of 10% presented an increase in total soluble solids content during storage time. However, the opposite was observed at a concentration of 30%.	[45]
Blueberry/ Blackberry	-Viability -Phenolic compounds -Organic acids -Antioxidant capacity -Anthocyanins -Sensory evaluation	*Lactiplantibacillus plantarum* BNCC337796 *Streptococcus thermophilus* CGMCC1.8748 *Bifidobacterium bifidum* CGMCC1.5090 Inoculum: 5 × 10^8^ Addition: free form and microencapsulated		**Viability:** Microbial counts of all three strains increased by approximately 0.4–0.7 Log _10_ CFU/mL in both juices after 48-h of fermentation. **Phenolic compounds:** Six phenolic acids were found in blackberry juice and seven in blueberry juice. Phenolic acid contents in blueberry and blackberry juice changed during the fermentation time. **Organic acids:** Citric acid was the prevalent organic acid observed in blueberry juice before fermentation, whereas tartaric acid was the major organic acid found in blackberry juice. Decreased pyruvic, shikimic, citric and malic acid levels were observed in both juices after fermentation, whereas lactic acid contents tended to increase. **Antioxidant capacity:** Overall, the addition of probiotics appears to have increased the antioxidant capacity of the juices. The highest antioxidant capacity was observed for juices fermented by *L. plantarum,* whereas the lowest one was recorded for juices fermented by *S. thermophilus.* **Sensory evaluation:** The sensory properties of fermented blackberry juices were different from those of fermented blueberry juices. Unlike blueberry juices, blackberry juices maintained a bright red color (a score of approximately 7.0). Aside from sourness and aftertaste, there were no significant differences in other sensory attributes among blackberry juice samples fermented by *L. plantarum, S. thermophilus* and *B. bifidum.* Blackberry juice fermented by *L. plantarum* recorded the highest scores for sour flavors (7.5) and acidity (7.4). They were followed by samples fermented by *B. bifidum* (6.5 and 6.6) and *S. thermophilus* (6.2 and 6.2). Sour (smell) and acidity (taste) flavor scores recorded for fermented blackberry juices were higher than those recorded for blueberry juice. On the other hand, sweetness score values recorded for all fermented blackberry juices (approximately 3.0) were lower than those observed for blueberry juices. Acids produced by *L. plantarum* decreased consumers’ purchase intention and acceptability towards blackberry juices, although they increased the acceptability of blueberry juices.	[28]

## Data Availability

Data supporting this study’s findings will be made available upon request to the corresponding author.
